# Barriers and enablers to help-seeking for common mental disorders among young people in low-income settings: Perspectives from Zimbabwe

**DOI:** 10.1371/journal.pone.0335963

**Published:** 2025-11-05

**Authors:** Rufaro Hamish Mushonga, Tarisai Concilia Bere, Rebecca Jopling, Franklin Glozah, Maria Anyorikeya, Tiny Tinashe Kamvura, Suzanne Dodd, Arnold Maramba, Denford Gudyanga, Benedict Weobong, Dixon Chibanda, Melanie Abas, Moses Kumwenda

**Affiliations:** 1 Institute of Psychiatry, Psychology and Neuroscience, King’s College London, London, United Kingdom,; 2 Department of Pathology, Kamuzu University of Health Sciences, Blantyre, Malawi,; 3 Department of Mental Health, Faculty of Medicine and Health Sciences, University of Zimbabwe, Harare, Zimbabwe,; 4 School of Public Health, University of Ghana, Accra, Ghana,; 5 Navrongo Health Research Centre, Research and Development Division, Navrongo, Ghana,; 6 The Friendship Bench, Zimbabwe, Harare; 7 Department of Social and Behavioural Sciences, University of Ghana, Accra, Ghana; 8 Faculty of Health, York University, School of Global Health, Toronto, Ontario, Canada; University of South Carolina, ZIMBABWE

## Abstract

Common Mental Disorders (CMDs), such as depression and anxiety are highly prevalent, particularly among young people globally. In Zimbabwe, contributing factors like poverty, unemployment, and the COVID-19 pandemic have exacerbated these challenges. Despite the pressing need for mental health support among young people, there remains a significant knowledge gap on barriers and enablers to help-seeking for CMDs among this demographic. This study addressed this gap by applying the Consolidated Framework for Implementation Research (CFIR) as an analytical framework to explore the unique factors influencing mental health help-seeking among young people in Zimbabwe.

**Methods:**

We utilised a qualitative research design and conducted 32 semi-structured interviews with young people (15–24 years) across high schools and the Friendship Bench (FB) in Harare between 20 December 2022 and 30 September 2023. Interviews were audiotaped and transcribed verbatim and then coded using an inductive approach to capture patterns grounded in participants’ experiences. Thematic analysis was utilised to develop relevant codes and identify relevant themes.

**Results:**

Nine themes were generated including six themes related to barriers (factors that hinder help-seeking for CMDs) and three themes related to enablers (factors that facilitate help-seeking for CMDs). Barriers identified include perceived stigma, privacy and confidentiality issues, unavailability of services, lack of awareness, financial challenges and lack of incentives. Enablers identified include raising awareness, implementing school based initiatives and enhancing accessibility and affordability of mental health services.

**Conclusion:**

This study revealed significant barriers and enablers to help-seeking for CMDs among young people in Zimbabwe. Addressing these multifaceted barriers and leveraging the identified enablers is key to creating supportive systems that encourage young people in low-resource settings to seek and engage with mental health services, ultimately improving their mental wellbeing and overall quality of life.

## Introduction

Common Mental Disorders (CMDs) such as depression and anxiety are highly prevalent particularly among young people, with rates affecting 10–20% of this demographic globally [1]. Additionally, CMDs account for 16% of the total global disease burden among young people [2]. In sub-Saharan Africa (SSA), young people aged 15–24 make up approximately 13.3% of the population, with an estimated 27% experiencing depression and 30% living with anxiety disorders [3,4]. Despite these high prevalence rates, access to mental healthcare, particularly in low-resource settings like Africa remains limited. Across the region, mental health service utilisation is critically low, with only 14 visits per 100 000 people annually compared with a global average of 1051 per 100 000 [5]. This gap is driven by severe shortages of trained personnel, limited availability of services, high out-of-pocket costs, low mental health literacy, and persistent stigma [6]. The mental health workforce is similarly constrained, with just 1.4 mental health workers per 100 000 people-far below the global average of 9-further limiting access to timely and effective care [5]. Treatments, when available, are often ineffective and not evidence-based, relying on approaches such as brief symptom-focused consultations, or traditional and spiritual healing without integration with biomedical care, leading to high disengagement rates [7,8]. Consequently, the proportion of Africans receiving treatment for mental health problems is among the lowest in the world.

In Zimbabwe, approximately 27% of young people aged 15–24 experience depression [9], though this is likely an underestimation. Contributing factors such as poverty, economic crisis, high unemployment, climate change, and the COVID-19 pandemic have created a complex web of challenges, exacerbating mental health issues among this vulnerable group [10–12]. Despite the evident need for mental health support, many young people in Zimbabwe do not seek help due to several barriers to accessing care. Previous research in Zimbabwe has predominantly focused on barriers to help-seeking for other health challenges, such as general non-communicable diseases [13], HIV treatment adherence [14,15], and barriers to mental health access among depressed postnatal women [16]. This leaves a significant gap in understanding the unique barriers and facilitators that influence mental health help-seeking among young people in Zimbabwe.

To address this gap, this study explored the factors that enable or hinder help-seeking for CMDs among young people in Zimbabwe, providing evidence to inform the design of contextually relevant and effective mental health interventions.

## Materials and methods

### Study design and setting

This study was conducted as part of formative work for the *African Youth in Mind* (Y-Mind) consortium, which aims to adapt and evaluate a stepped care intervention for youth aged 15–24 experiencing depression or anxiety. The intervention is delivered through a task-shifting approach, engaging non-specialist providers in Ghana and Zimbabwe. In Ghana, the Y-Mind intervention targets young people aged 15–18 enrolled in high schools, while in Zimbabwe, it is designed for young people aged 15–24, delivered across both school and community health settings.

A qualitative research design was employed for this study. Data collection took place between 20 December 2022 and 30 September 2023. The study participants included students from selected high schools in Harare’s high density suburbs, as well as young people involved with the Friendship Bench (FB) program. This included those who had received problem-solving therapy (PST) for CMDs-referred to as *clients*- and those trained to deliver PST, also known as *buddies* or peer counsellors. The Friendship Bench is an evidenced-based intervention developed in Zimbabwe to bridge mental health treatment gap. It uses a cognitive behavioural therapy (CBT) based approach at primary care level to address “kufungisisa”-the local word closest to depression (literally, “thinking too much” in English). Uniquely, the FB uses ‘grandmothers’ and ‘youth buddies’ (peer counsellors) to deliver the therapy [17,18]. These grandmothers are community volunteers, without any prior medical or mental health experience, and are trained to counsel patients usually for six structured 45-minute sessions, on wooden benches within the grounds of clinic in a discrete area [19]. In reporting findings of this study, we adhered to the Consolidated criteria for REporting Qualitative research checklist (COREQ checklist).

### Analytical framework

This study employed the Consolidated Framework for Implementation Research (CFIR) [20] as a guiding analytical framework to explore the barriers and enablers influencing help-seeking for CMDs among young people in Zimbabwe. The CFIR is a widely used framework for identifying factors that shape implementation outcomes across five domains: ‘Inner Setting’, ‘Outer Setting’, ‘Intervention Characteristics’, ‘Implementation Process’, and ‘Characteristics of Individuals’ [20,21]. In this study, only the ‘Inner Setting’ and ‘Implementation Process’ were applied as data from other domains were limited. The ‘Inner Setting’ captures the institutional, structural, political, and contextual factors such as leadership, communication, and available resources that can influence intervention delivery [22]. The ‘Implementation Process’ domain refers to the specific activities and strategies used to implement the intervention [16].

### Inclusion and exclusion criteria

Participants were included if they were aged between 15 and 24 years, capable of providing consent or assent. Those with lived experience had received formal care and were identified through the FB programme. This group included individuals who had responded to PST, defined as achieving a score below 8 on the Shona Symptom Questionnaire (SSQ-14) at their exit session. The SSQ-14 is a culturally validated depression screening tool developed specifically for Zimbabwe and neighbouring regions of sub-Saharan Africa [23]. Participants were excluded if they had an active major mental health condition, determined by a previous clinical diagnosis of a severe mental health condition that could impair study participation. Individuals who were actively suicidal, assessed using the P4 screener which assesses suicide risk through the “4P’s”: past suicide attempts, a plan, probability of completing suicide, and preventive factors [24]-or those with an advanced physical illness or impairment identified during informed consent procedures were also excluded.

### Sampling and recruitment

Participants aged 15–24 years were purposively sampled from two groups: high school students in Harare’s high-density suburbs and young people affiliated with the FB. For high school students, permission was obtained from the Ministry of Primary and Secondary Education and the participating schools. School authorities helped identify students meeting the inclusion criteria, and information sheets were provided to participants. Those < 18 years old were given parental information sheets and assent forms to take home for their parents or guardians to review and sign, after which the students provided their own assent. Those ≥18 years old were given information sheets and informed consent forms to sign. At the selected schools, those who returned signed forms were 10 students (5 males, 5 females). For the FB group, recruitment included trained peer counsellors (buddies) and clients-young people with lived experience of depression who had completed Problem-Solving Therapy (PST) within the past 12 months. Buddies were identified by FB supervisors based on their experience delivering PST, while clients were identified from the FB records. Eligible clients were first approached by their FB care provider to assess interest, after which research assistants provided full study information and obtained written consent/assent. The FB sample included 7 buddies (3 males, 4 females) and 15 clients (6 males, 9 females). Data collection continued until thematic saturation, and the final study sample comprised 32 participants (14 males, 18 females).

A detailed summary of participant characteristics is presented in Table 1.

**Table 1:** Demographic characteristics of participants.

PID	Category	Age	Sex
P1	Friendship Bench Buddy	24	Male
P2	Friendship Bench Buddy	22	Male
P3	Friendship Bench Buddy	22	Female
P4	Friendship Bench Buddy	22	Female
P5	Friendship Bench Buddy	23	Female
P6	Friendship Bench Buddy	24	Female
P7	Friendship Bench Buddy	22	Male
P8	Friendship Bench Client	17	Female
P9	Friendship Bench Client	23	Female
P10	Friendship Bench Client	21	Female
P11	Friendship Bench Client	22	Female
P12	Friendship Bench Client	23	Female
P13	Friendship Bench Client	22	Male
P14	Friendship Bench Client	19	Female
P15	Friendship Bench Client	20	Female
P16	Friendship Bench Client	20	Male
P17	Friendship Bench Client	19	Male
P18	Friendship Bench Client	22	Male
P19	Friendship Bench Client	22	Female
P20	Friendship Bench Client	18	Male
P21	Friendship Bench Client	22	Female
P22	Friendship Bench Client	20	Male
P23	High School Student	17	Female
P24	High School Student	18	Male
P25	High School Student	17	Female
P26	High School Student	17	Male
P27	High School Student	17	Female
P28	High School Student	18	Male
P29	High School Student	18	Female
P30	High School Student	18	Male
P31	High School Student	18	Male
P32	High School Student	18	Female

### Data collection

To gather data, we used semi-structured interviews with a core list of open-ended questions. Each session, which lasted between 40–60 minutes, was conducted by 3 Research Assistants (RAs), including one of the co-authors (AM), all of whom held at least a bachelor’s degree in social sciences. The RAs, bilingual in Shona and English, had a prior experience in qualitative data collection, and received additional training and ongoing supervision from their supervisor (TTK). Transcripts underwent quality checks, followed by pseudo- anonymization to ensure the confidentiality of participants’ identities. All interviews were audio-recorded, transcribed verbatim and translated from Shona (the local language) to English by experienced RAs. Finalised transcripts were securely stored on Microsoft SharePoint, with access limited to study staff trained in Good Clinical Practice (GCP). Audio recordings were deleted following transcription and verification.

### Data analysis

Data were analysed using NVivo 14 following an inductive thematic analysis to capture patterns grounded in participants’ experiences. Nine co-authors (RHM, TCB, RJ, FG,MA, TTK,SD, AM and DG) independently coded each transcript line-by-line, generating codes directly from the data. Codes were refined through bi-weekly team discussions and final themes were agreed through iterative review involving a senior researcher (MK). After inductive coding process, the CFIR framework was then applied. All five CFIR domains were initially considered, but only ‘Inner Setting’ and ‘Implementation Process’ consistently aligned with the data. Codes were examined and then fitted under relevant CFIR constructs based on conceptual alignment. In some cases, codes mapped naturally to existing constructs, while in others, placement required interpretive judgement. This approach ensured theory-informed interpretation while retaining fidelity to participants’ perspectives.

### Ethical considerations

Approval for this study was obtained from the Medical Research Council of Zimbabwe (Ref: MRCZ/A/2965) and the Kings College London Research Ethics (Ref: HR/DP-21/22–32917). Permission was also sought from the Ministry of Primary and Secondary Education for selected high schools in Harare’s high-density suburbs. Participation was voluntary, and written informed consent/assent was obtained from all participants. Those ≥ 18 years old signed their own consent forms, while those < 18 years old provided assent alongside parental or guardian consent. Confidentiality and anonymity were ensured by omitting identifying information from transcripts and securely storing data. All participants received USD $10 to cover transport and related costs.

### Findings

From our data, we identified nine distinct themes that mapped onto the CFIR domains of ‘Inner Setting’ and ‘Implementation Process’. Six themes were designated as barriers and three as enablers. Table 2 shows major themes generated across CFIR domains and constructs.

**Table 2:** Themes generated across CFIR domains and constructs.

CFIR Domain	CFIR Construct	Implementation Determinant	Theme
**Inner Setting**	Culture	Barrier	• Perceived stigma • Privacy and confidentiality issues
	Available resources	Barrier	• Unavailability of services • Financial challenges
	Organisational incentives and rewards	Barrier	• Lack of incentives
	Access to knowledge and information	Barrier	• Lack of awareness
**Inner Setting**	Access to knowledge and information	Enabler	• Raising awareness
	Available resources	Enabler	• Enhancing accessibility and affordability of mental health services
**Implementation Process**	Engaging	Enabler	• Implement school based initiatives

### Barriers to help-seeking

Our findings highlight several barriers that limit young people’s engagement with mental health services in Zimbabwe. All barriers were associated with the ‘Inner Setting’ domain of the CFIR framework and cluster around cultural factors, limited resources, lack of incentives, and inadequate access to knowledge and information.

### Inner setting-culture

#### Perceived stigma.

A central cultural barrier identified in this study was perceived stigma. This stigma is rooted in deep-seated and widely shared social beliefs that view mental health issues as shameful, or morally suspect, leading to fear of judgement, social rejection, and feelings of shame and guilt.

Consequently, many opt to suffer in silence rather than seek help. One young participant with no lived experience of a mental health condition shared:

*Others would be shy. Let’s say she was impregnated or raped, she would think that if they tell the teacher, she* [the teacher] *would tell others and they will laugh at them-*P32

Another participant reflected:

*I think fear drives them* [young people] *away. Because they would be thinking that when they share their problems, people would laugh at them or belittle them-*P1

These excerpts underscore how perceived stigma operates independently of actual disclosure or engagement with services. It is anticipatory and internalised, where young people fear the societal consequences of being associated with mental health problems, even in hypothetical scenarios. As such, perceived stigma is not about actual breaches to confidentiality, but about broad social attitudes that cast mental health conditions as a source of judgement and shame. This results in a culture of silence, where many opt to suffer alone rather than risk being labelled or laughed at.

#### Privacy and confidentiality issues.

Privacy and confidentiality concerns are also significant cultural barriers to help-seeking among young people. Participants fear that sensitive information might be disclosed without consent, driven by past breaches or cultural and institutional norms. This fear was especially pronounced among young people with lived experience of mental health conditions, affecting their trust and willingness to seek help. One participant shared their hesitation to confide in care providers due to concerns about trust and discretion:

*Sometimes you think, ‘if I go and ask this person* [care provider], *what if they go and talk about me saying so and so did this?’ It is not a good thing because you will be thinking that you have told someone that you trust, and then that person goes and tells someone-*P9

Similarly, another participant highlighted how these fears are compounded by feelings of embarrassment and potential judgement:

*Let me use myself as an example. It wasn’t easy for me to just come to the clinic and head straight to the grandmother* [lay healthcare provider] *in the black t-shirt. I couldn’t just walk up to her and tell her that I have an STI? It’s not easy. So, fear and embarrassment take over because you think that once you share, they will spread the news and you will become talk of the day or a laughingstock-*P22

These fears are exacerbated in public or family settings, where young people feel especially vulnerable to judgement and misunderstanding. Many shared how discussing mental health struggles in front of family members or acquaintances felt particularly distressing. For instance, another participant with a lived experience of a mental health condition recounted their experience:

*Gogo* [grandmother] *comes to the front and starts saying if you have anxiety and depression come to the bench. And you’re there with your mother or someone that you know, and the next thing you know, they are asking what you are really depressed about-*P15

This public call-out approach, coupled with intrusive questions, seems to undermine the sense of privacy and confidentiality necessary for young people to feel safe sharing their mental health struggles. The discomfort caused by these situations often prevents them from seeking help altogether leaving many to suffer in silence rather than becoming the subject of embarrassment. Unlike perceived stigma, which reflects internalised societal beliefs, privacy and confidentiality concerns are relational, situational, and context specific. They reflect a lack of trust in individuals or systems to keep disclosures private, especially in close-knit or high-surveillance environments such as homes, clinics, or schools.

### Inner setting-available resources

#### Unavailability of mental health services.

The limited availability of mental health services in Zimbabwe is significantly impacting young people’s ability to seek help for mental health conditions. This leaves a critical gap in accessibility for most young people in dire need of mental health services, especially those in underserved locations. One young individual with a history of depression expressed their frustration:

*I didn’t do the other sessions because where I stay the Friendship Bench organization is not there-*P16

Another participant with a lived experience shared similar sentiments:

*People do not know about FB. Look at this place right now almost everyone is facing something. It’s just the issue of your location which is only at hospitals or clinics-*P10

Beyond FB, participants stressed that young people in rural areas face even greater barriers because of the absence of any structured mental health services. As one participant put it:

*In most rural areas, there are no mental services offered to young people and many are suffering-*P27

These accounts highlight that the unavailability of both government and NGO-run mental health services, compounded by their concentration in urban areas, significantly constrains young people’s ability to seek help.

#### Financial challenges.

Also, financial barriers, particularly those linked to the costs of digital access, emerged as significant obstacles to young people’s engagement with mental health care. While services such as the FB have introduced online PST via WhatsApp as an alternative to in-person sessions, many young people reported being unable to take full advantage of these innovations. For instance, the high cost of mobile data, combined with limited access to smartphones or stable internet, create substantial inequities in who could benefit from these services. A peer counsellor described this challenge they face in trying to support young people online:

*I always try to follow them* [YP with mental health challenges] *on WhatsApp and you find that some do not even reply because they do not have WhatsApp bundles and this problem is genuine-*P4

Similarly, a participant with lived experience of mental health challenge described how financial constraints disrupted continuity of care:

*I didn’t continue with the online therapy. I ran out of data bundles, so I didn’t stay in touch with the counsellor-*P8

These accounts underscore that financial challenges in this context are largely technology-driven, limiting both engagement and sustained follow-up in digital mental health services.

### Inner setting-incentives and rewards

#### Lack of incentives.

Our study found that lack of incentives can hinder young people’s willingness to seek help and participate fully in mental health services. For most young people, who are often preoccupied with pressing economic needs, services that do not provide immediate or material benefits may seem irrelevant or low priority. An FB peer counsellor recounted common reactions from young individuals with mental health issues:

*They will always ask, ‘Counselling sessions last 45 minutes to an hour-what will you give me in that time?’-*P6

Another peer counsellor described how the perceived lack of concrete outcomes undermines the value of counselling in the eyes of these young people:

*Because you’ve talked to them, and you don’t offer something tangible, they feel they don’t need you. They tell you, ‘Since you can’t really solve my problem, I don’t think I still need to be talking to you. I need to look for something to do to get money-*P2

These perspectives highlight the need to better align mental health interventions with the socio-economic realities and expectations of young people-particularly those not yet engaged in understanding or managing mental health issues.

### Inner setting- access to knowledge and information

#### Lack of awareness.

Another significant barrier preventing young people in Zimbabwe from seeking mental health support is the pervasive lack of awareness about CMDs and available support services. This information gap fosters harmful misconceptions, with many linking mental health conditions to supernatural causes such as witchcraft, evil spirits, or satanism. For instance, one participant with a history of mental illness described the fear these beliefs instilled:

*When I spoke about my situation, people said you might encounter satanism if you receive everything that is given to you-*P13

Another participant expressed similar concerns:

*It’s like if you hear that these people* [mental health care service providers] *are into satanism, this would instil fear of being initiated into satanism-*P15

In addition to cultural misconceptions, a general lack of information about the availability of mental health services further hinders access. For instance, some only learned about specific programmes such as the FB by chance, often during visits to clinics for unrelated health issues. One participant with a history of mental health challenge shared:

*What hinders many young people from accessing these mental health services is lack of knowledge about organisations that can help in that regard. Just like Friendship Bench which offers mental health services, I only got to learn about it when I came to the clinic seeking medical attention. So lack of information can cause somebody not to access these services-*P20

These sentiments reflects broader gaps in knowledge about mental health and availability of support services in Zimbabwe. Targeted awareness campaigns are therefore urgently needed to dispel myths and improve access to accurate information about mental health and existing services.

### Enablers to help-seeking

In our study, identified enablers for help-seeking for CMDs fall within the ‘Inner Setting’ and ‘Implementation Process’ domains of the CFIR framework.

### Inner setting-access to knowledge and information

#### Raising awareness.

Study participants consistently highlighted raising awareness as a key enabler to help-seeking for mental health conditions. They emphasised that raising awareness empowers them with knowledge, dispel misconceptions, and create a supportive environment conducive to seeking mental health support. For instance, one participant with a history of a mental health condition noted:

*I think what will help us as young people is awareness. Awareness on where to go for assistance when we are experiencing mental health challenges-*P17

Participants also stressed the need to redefine perceptions of mental health, moving away from stereotypes of insanity and emphasizing general mental well-being. Efforts by government and NGOs were recognised as key in this regard. One participant alluded:

*It’s important that the NGOs or the government should commit themselves and try to remove that idea that when people talk of mental health-related issues it’s not only about being insane or crazy and stuff, but mental health refers to the general well-being of the mind-*P30

Additionally, utilising various media platforms such as radios, TVs and social media were identified as effective channels to raise awareness.

*Advertising on TVs would be better. And then on social media maybe people they are on Facebook, Instagram so on. Parents will know it from all those platforms-*P24

Lastly, involving influential figures such as artists was highlighted as an impactful strategy, leveraging relatability to engage young people effectively.

*Invite artists like Winky D probably when you want to talk about mental health, and whatever message he has they* [young people] *will listen-*P7

### Inner setting-available resources

#### Enhancing accessibility and affordability of mental health services.

Enhancing accessibility and affordability of mental health services was also recognised as a crucial enabler for mental health support. Participants stressed the significance of overcoming physical barriers by providing for example adequate infrastructure, ensuring that counselling sessions remain accessible even during adverse weather conditions. Since FB sessions are often conducted outdoors on benches without overhead cover, they are particularly vulnerable to disruption. One FB peer counsellor explained:

*You know when the weather subsequently changes in the midst of the session and it starts raining, the session will be disrupted and the client will just disappear. That’s a big challenge, so at least if shades are put there it will be okay to work especially in rain season-*P5

Others proposed establishment of mental health services in multiple locations to address the need for convenient access. For instance, a participant with a lived experience of a mental health condition suggested:

*If you have several mental health services in different locations it could improve on convenience for the people to access these services-*P9

Also, affordability was emphasised as a key enabler, though participants were not referring to counselling fees directly, as the FB services are offered free of charge. Instead, concerns centred on the indirect costs associated with accessing care, such as transport expenses, or the costs of follow-up medical support. These financial pressures act as deterrents, with some participants describing how people give up when faced with such barriers. Thus participants advocated for reduced or free mental health services to alleviate financial constraints. One participant explained:

*Maybe make it a bit more affordable and accessible. Because for most people feel like they hit a brick wall and they make a U-turn-*P29

### Implementation process-engaging

#### Implementing school based initiatives.

Study participants proposed implementing school-based initiatives as a key enabler to mental health support. Suggestions revolves around the establishment of health clubs dedicated to address mental health issues. According to participants, the clubs would provide an open and dynamic space for open discussions and collective problem solving, particularly for mental health issues. However, to ensure inclusivity and overcome potential barriers related to reluctance or shyness, suggestions like the incorporation of suggestion boxes, for anonymously submitting grievances was highlighted.

*At high school level I think maybe establishing a health club that specializes in dealing with the issues of the students. As a club we can meet and talk of the issues. Maybe we could say that we have our suggestion box whereby the students fill their grievances that they want to be addressed. Because some might be shy to talk of mental health issues-*P28

Additionally, engagement in sporting activities was stressed as a means to enhance distraction from life stressors and fostering peer interaction and support.

*There is need for activities like ball games. If you go there, you become occupied then stress will be reduced because you will not be concentrating on life issues. You can also mix with others-*P25

Lastly, participants also stressed the importance of school-based income generating-initiatives in facilitating mental health support among vulnerable students. They suggested that creating opportunities within schools, such as vocational workshops, could both ease financial stressors that often contribute to depression and promote positive mental health through skills development and purposeful engagement. For instance, one student explained:

*There is need to regenerate our metal work technology workshop through welding and iron technology. Maybe they* [students] *can make tools and utensils that they may sell and earn income so that it may help those who are financially unstable. And also the idea of working it helps the mind stay focused on what you are doing rather than thinking of the problems you have at home-*P25

This perspective highlights how schools can serve not only as academic institutions but also as supportive environments that foster resilience, reduce financial stress, and strengthen students’ overall mental well-being.

## Discussion

This study explored barriers and enablers influencing help-seeking for CMDs among young people in Zimbabwe, using the CFIR framework as an analytical framework. Six barrier-related themes and three enabler -related were identified, providing critical insights into young people’s help-seeking behaviours. Barriers were predominantly located within the ‘Inner Setting’ domain, particularly in relating to cultural norms, structural characteristics, available resources, organisational incentives and access to knowledge and information. Enablers withing the ‘Inner Setting’ domain included interventions that raise awareness, improve accessibility and affordability of mental health services, and promoting school-based initiatives.

Perceived stigma was a central issue, driven by deeply ingrained societal stereotypes about mental illness. This stigma fosters secrecy, reluctance to seek help, and avoidance of support services. These findings align with previous research on perceived stigma, which describes the internalisation of negative societal stereotypes by individuals with mental illness [25,26]. Perceived stigma involves internalised beliefs that people with mental illness are incompetent or dangerous, which can lead to negative self-evaluations, self-prejudice, and harmful cognitive and emotional effects. Therefore, young individuals often internalise emotions such as fear, guilt, and shame [25,27,28], leading to secrecy and reduced help-seeking behaviours [29,30]. Privacy and confidentiality issues further compound this reluctance, as fears of judgement and breaches of trust discourage young people from seeking support. These results align with prior research which suggests that self-stigma can lead young people to seek help from trusted sources [31,32]. Together, these studies highlight that privacy and confidentiality are critical considerations in the design and delivery of youth mental health services [30,32].

Young people’s limited understanding of mental health, from both psychosocial and biomedical perspectives, particularly in low-resource settings further discourages them from seeking mental health support [33,34]. These findings align with previous studies, where misconceptions about mental health lead to inappropriate treatment-seeking behaviours [33–35]. Also, these misconceptions often result in mistrust of interventions and delay access to appropriate care [36]. Lack of information about available services further prevents young people from seeking mental health support, as shown in previous research where insufficient knowledge about mental health contributes to resistance to interventions [20,35].

In addition, financial challenges and limited accessibility were significant barriers within the ‘Inner Setting’ domain of the CFIR framework. Economic difficulties disproportionately affect young people, limiting their ability to access in-person services or afford data for online therapy sessions. Financial constraints are well-documented as obstacles to help-seeking in low-resource settings [32,37–40]. Additionally, the lack of tangible benefits for participating in counselling sessions diminishes young people’s engagement with mental health services, a challenge similarly noted in studies where financial incentives were identified as motivators for service utilisation [32]. Accessibility issues are further exacerbated by inadequate infrastructure and the absence of mental health services in underserved locations, particularly rural areas, where high travel costs and poor infrastructure hinder access [32]. This highlights the need for more inclusive service delivery models that account for socioeconomic determinants such as poverty, transport barriers, and digital access when designing mental health interventions.

Despite these barriers identified, several enablers emerged within the ‘Inner Setting’ and ‘Implementation Process’ domains of the CFIR framework. Within the ‘Inner Setting’, raising awareness was identified as a pivotal factor. Awareness empowers young people and their caregivers with knowledge, dispels misconceptions, and fosters supportive environments conducive to help-seeking. Broader awareness initiatives, including media campaigns and the involvement of influential figures, have been effective in improving mental health literacy, particularly in low-resource settings [34,36,41,42].

Improving access and affordability was another key enabler within the ‘Inner Setting’ domain. Participants emphasised the importance of adequate infrastructure, expanding services such as the FB to underserved areas, and reducing indirect costs of care. These findings align with previous studies, which highlight the critical role of expanding primary care facilities and improving proximity to mental health services in low-income settings [43–45].

Within the ‘Implementation Process’ domain, school-based initiatives were identified as effective strategies to promote help-seeking. Activities such as health clubs, sports, and income-generating projects provide opportunities for peer support, collective problem-solving, and skills development, while also addressing socioeconomic determinants such as financial stress and lack of purposeful engagement [46,47]. For example, sports activities enhance social cohesion, provide structured engagement, whereas income-generating initiatives help financially vulnerable students to reduce economic stressors that contribute to mental health problems.

Despite these insights, several limitations must be acknowledged. First, the study focused on CMDs among young people aged 15–24, which may limit the transferability of findings to other mental health conditions, such as psychosis, or substance use disorders. Second, geographical coverage was limited to high-density suburbs in Harare, excluding rural and other urban contexts, restricting the generalisability of findings to broader populations. Third, data collection spanned over nine months, introducing potential temporal bias, as participants’ experiences and perceptions may have changed over this period. Fourth, the CFIR framework was applied retrospectively during data analysis, rather than being integrated a priori into the study design, which may have constrained the identification of certain implementation determinants. Fifth, while our study focused on the ‘Inner Setting’ and ‘Implementation Process’ domains, other domains (e.g., Outer Setting, Characteristics of Intervention, Intervention Characteristics) were deliberately excluded as they didn’t generate sufficient data, potentially limiting the breadth of insights. Finally, the sample was only restricted to young individuals aged 15–24, and this might have excluded unique perspectives from other age groups, and influencers such as caregivers, teachers, religious leaders etc. Incorporating these views in future research would provide a more holistic understanding of the social and structural factors shaping mental health help-seeking among young people in Zimbabwe.

Taken together, the implications of this study are significant for both policy and practice in Zimbabwe. Improving youth access to mental health services in Zimbabwe requires a multifaceted approach that addresses cultural stigma, enhances confidentiality and trust, expands service availability, and considers broader socioeconomic determinants. Efforts to reduce stigma must go beyond surface-level messaging and instead employ culturally sensitive, community-driven approaches that acknowledge local beliefs and contexts. Awareness campaigns co-developed with young people, religious leaders, and traditional authorities can help dispel misconceptions and foster openness around mental health. Schools represent a vital platform for early intervention and literacy-building. Integrating mental health into the formal curriculum and extracurricular activities such as peer-led clubs, sports, and income-generating projects can enhance psychosocial support, encourage help-seeking, and reduce distress among students. At the same time, expanding access to affordable mental health services is critical. This includes decentralising care through community-based models, mobile outreach, and digital platforms, particularly in underserved rural areas where young people face compounded barriers. Furthermore, strengthening mental health literacy among both young people and influential community members such as parents, teachers, and faith leaders will help shift norms and increase support for those experiencing mental health challenges. Crucially, young people should not only be the focus of interventions but also active participants in shaping and evaluating them to ensure relevance and impact. By acting on these recommendations, Zimbabwe can take meaningful steps toward a more responsive and youth-centred mental health system.

## Conclusion

This study highlights significant barriers and enablers influencing help-seeking for common mental disorders (CMDs) among young people in Zimbabwe, offering insights that can inform the development and optimisation of mental health interventions in low-resource settings. While our findings reveal critical barriers such as perceived stigma, cultural misconceptions, financial constraints, and accessibility challenges, they also underscore key enablers including awareness-raising efforts, improvements to accessibility and affordability and school-based initiatives. Addressing these multifaceted barriers and leveraging the identified enablers is key in creating supportive systems that encourage young people in low-resource settings to seek and engage with mental health services, ultimately improving their mental wellbeing and overall quality of life.

## Supporting information

S1 File Inclusivity-in-global-research-questionnaire. (DOCX)

S2 File Interview guide-No lived experience. (DOCX)

S3 File Peer counsellors interview guide. (DOCX)

S4 File Updated CFIR domain and construct definitions. (DOCX)
