# Supplementary material for: Barriers and enablers to help-seeking for common mental disorders among young people in low-income settings: Perspectives from Zimbabwe
Source: PLoS One. 2025 Nov 5;20(11):e0335963. doi: 10.1371/journal.pone.0335963 (PMC12588522; doi:10.1371/journal.pone.0335963)
Supplement: S4 File — (DOCX) [file pone.0335963.s004.docx]

**Updated CFIR domain and construct definitions**

| **I. Innovation domain**  ***Innovation:*** The “thing” being implemented, e.g., a new clinical treatment, educational program, or city service  ***Project Innovation:*** [Document the innovation being implemented, e.g., innovation type, innovation core vs. adaptable components, using a published reporting guideline. Distinguish the innovation (the “thing” that continues when implementation is complete) from the implementation process and strategies used to implement the innovation (activities that end after implementation is complete). | |
| --- | --- |
| **Construct name** | **Construct definition**  *The degree to which:* |
| A. Innovation Source | The group that developed and/or visibly sponsored use of the innovation is reputable, credible, and/or trustable |
| B. Innovation Evidence Base | The innovation has robust evidence supporting its effectiveness |
| C. Innovation Relative Advantage | The innovation is better than other available innovations or current practice |
| D. Innovation Adaptability | The innovation can be modified, tailored, or refined to fit local context or needs |
| E. Innovation Trialability | The innovation can be tested or piloted on a small scale and undone |
| F. Innovation Complexity | The innovation is complicated, which may be reflected by its scope and/or the nature and number of connections and steps |
| G. Innovation Design | The innovation is well designed and packaged, including how it is assembled, bundled, and presented |
| H. Innovation Cost | The innovation purchase and operating costs are affordable |
| **II. Outer Setting domain**  ***Outer Setting:*** The setting in which the Inner Setting exists, e.g., hospital system, school district, state. There may be multiple Outer Settings and/or multiple levels within the Outer Setting, e.g., community, system, state  ***Project Outer Setting(s):*** [Document the actual Outer Setting in the project, e.g., type, location, and the boundary between the Outer Setting and the Inner Setting.] | |
| **Construct name** | **Construct definition**  *The degree to which:* |
| A. Critical Incidents | Large-scale and/or unanticipated events disrupt implementation and/or delivery of the innovation |
| B. Local Attitudes | Sociocultural values (e.g., shared responsibility in helping recipients) and beliefs (e.g., convictions about the worthiness of recipients) encourage the Outer Setting to support implementation and/or delivery of the innovation |
| C. Local Conditions | Economic, environmental, political, and/or technological conditions enable the Outer Setting to support implementation and/or delivery of the innovation |
| D. Partnerships & Connections | The Inner Setting is networked with external entities, including referral networks, academic affiliations, and professional organization networks |
| E. Policies & Laws | Legislation, regulations, professional group guidelines and recommendations, or accreditation standards support implementation and/or delivery of the innovation |
| F. Financing | Funding from external entities (e.g., grants, reimbursement) is available to implement and/or deliver the innovation |
| G. External Pressure | External pressures drive implementation and/or delivery of the innovation  *Use this construct to capture themes related to External Pressures that are not included in the subconstructs below* |
| 1. Societal Pressure | Mass media campaigns, advocacy groups, or social movements or protests drive implementation and/or delivery of the innovation |
| 2. Market Pressure | Competing with and/or imitating peer entities drives implementation and/or delivery of the innovation |
| 3. Performance Measurement Pressure | Quality or benchmarking metrics or established service goals drive implementation and/or delivery of the innovation |
| **III. Inner Setting domain**  ***Inner Setting:*** The setting in which the innovation is implemented, e.g., hospital, school, city. There may be multiple Inner Settings and/or multiple levels within the Inner Setting, e.g., unit, classroom, team  ***Project Inner Setting(s):*** [Document the actual Inner Setting in the project, e.g., type, location, and the boundary between the Outer Setting and the Inner Setting.] | |
| **Construct name** | **Construct definition**  *The degree to which:* |
| *Note:* | *Constructs A – D exist in the Inner Setting regardless of implementation and/or delivery of the innovation, i.e., they are persistent general characteristics of the Inner Setting* |
| A. Structural Characteristics | Infrastructure components support functional performance of the Inner Setting  *Use this construct to capture themes related to Structural Characteristics that are not included in the subconstructs below* |
| 1. Physical Infrastructure | Layout and configuration of space and other tangible material features support functional performance of the Inner Setting |
| 2. Information Technology Infrastructure | Technological systems for tele-communication, electronic documentation, and data storage, management, reporting, and analysis support functional performance of the Inner Setting |
| 3. Work Infrastructure | Organization of tasks and responsibilities within and between individuals and teams, and general staffing levels, support functional performance of the Inner Setting |
| B. Relational Connections | There are high quality formal and informal relationships, networks, and teams within and across Inner Setting boundaries (e.g., structural, professional) |
| C. Communications | There are high quality formal and informal information sharing practices within and across Inner Setting boundaries (e.g., structural, professional) |
| D. Culture | There are shared values, beliefs, and norms across the Inner Setting  *Use this construct to capture themes related to Culture that are not included in the subconstructs below* |
| 1. Human Equality-Centeredness | There are shared values, beliefs, and norms about the inherent equal worth and value of all human beings |
| 2. Recipient-Centeredness | There are shared values, beliefs, and norms around caring, supporting, and addressing the needs and welfare of recipients |
| 3. Deliverer-Centeredness | There are shared values, beliefs, and norms around caring, supporting, and addressing the needs and welfare of deliverers |
| 4. Learning-Centeredness | There are shared values, beliefs, and norms around psychological safety, continual improvement, and using data to inform practice |
| *Note:* | *Constructs E – K are specific to the implementation and/or delivery of the innovation* |
| E. Tension for Change | The current situation is intolerable and needs to change |
| F. Compatibility | The innovation fits with workflows, systems, and processes |
| G. Relative Priority | Implementing and delivering the innovation is important compared to other initiatives |
| H. Incentive Systems | Tangible and/or intangible incentives and rewards and/or disincentives and punishments support implementation and delivery of the innovation |
| I. Mission Alignment | Implementing and delivering the innovation is in line with the overarching commitment, purpose, or goals in the Inner Setting |
| J. Available Resources | Resources are available to implement and deliver the innovation  *Use this construct to capture themes related to Available Resources that are not included in the subconstructs below* |
| 1. Funding | Funding is available to implement and deliver the innovation |
| 2. Space | Physical space is available to implement and deliver the innovation |
| 3. Materials & Equipment | Supplies are available to implement and deliver the innovation |
| K. Access to Knowledge & Information | Guidance and/or training is accessible to implement and deliver the innovation |
| **IV. Individuals domain**  ***Individuals:*** The roles and characteristics of individuals | |
| **Roles subdomain**  ***Project Roles:*** [Document the roles applicable to the project and their location in the Inner Setting or Outer Setting.] | |
| **Construct name** | **Construct definition** |
| A. High-level Leaders | Individuals with a high level of authority, including key decision-makers, executive leaders, or directors |
| B. Mid-level Leaders | Individuals with a moderate level of authority, including leaders supervised by a high-level leader and who supervise others |
| C. Opinion Leaders | Individuals with informal influence on the attitudes and behaviors of others |
| D. Implementation Facilitators | Individuals with subject matter expertise who assist, coach, or support implementation |
| E. Implementation Leads | Individuals who lead efforts to implement the innovation |
| F. Implementation Team Members | Individuals who collaborate with and support the Implementation Leads to implement the innovation, ideally including Innovation Deliverers and Recipients |
| G. Other Implementation Support | Individuals who support the Implementation Leads and/or Implementation Team Members to implement the innovation |
| H. Innovation Deliverers | Individuals who are directly or indirectly delivering the innovation |
| I. Innovation Recipients | Individuals who are directly or indirectly receiving the innovation |
| **Characteristics subdomain**  ***Project Characteristics:*** [Document the characteristics applicable to the roles in the project based on the COM-B system [[29](https://implementationscience.biomedcentral.com/articles/10.1186/s13012-022-01245-0#ref-CR29)] or role-specific theories.] | |
| **Construct name** | **Construct definition:**  *The degree to which:* |
| A. Need | The individual(s) has deficits related to survival, well-being, or personal fulfillment, which will be addressed by implementation and/or delivery of the innovation |
| B. Capability | The individual(s) has interpersonal competence, knowledge, and skills to fulfill Role |
| C. Opportunity | The individual(s) has availability, scope, and power to fulfill Role |
| D. Motivation | The individual(s) is committed to fulfilling Role |
| **V. Implementation Process domain**  ***Implementation Process:*** The activities and strategies used to implement the innovation  ***Project Implementation Process:*** [Document the implementation process framework and/or activities and strategies being used to implement the innovation. Distinguish the implementation process used to implement the innovation (activities that end after implementation is complete) from the innovation (the “thing” that continues when implementation is complete). | |
| **Construct name** | **Construct definition:**  *The degree to which individuals:* |
| A. Teaming | Join together, intentionally coordinating and collaborating on interdependent tasks, to implement the innovation |
| B. Assessing Needs | Collect information about priorities, preferences, and needs of people  *Use this construct to capture themes related to Assessing Needs that are not included in the subconstructs below* |
| 1. Innovation Deliverers | Collect information about the priorities, preferences, and needs of deliverers to guide implementation and delivery of the innovation |
| 2. Innovation Recipients | Collect information about the priorities, preferences, and needs of recipients to guide implementation and delivery of the innovation |
| C. Assessing Context | Collect information to identify and appraise barriers and facilitators to implementation and delivery of the innovation |
| D. Planning | Identify roles and responsibilities, outline specific steps and milestones, and define goals and measures for implementation success in advance |
| E. Tailoring Strategies | Choose and operationalize implementation strategies to address barriers, leverage facilitators, and fit context |
| F. Engaging | Attract and encourage participation in implementation and/or the innovation  *Use this construct to capture themes related to Engaging that are not included in the subconstructs below* |
| 1. Innovation Deliverers | Attract and encourage deliverers to serve on the implementation team and/or to deliver the innovation |
| 2. Innovation Recipients | Attract and encourage recipients to serve on the implementation team and/or participate in the innovation |
| G. Doing | Implement in small steps, tests, or cycles of change to trial and cumulatively optimize delivery of the innovation |
| H. Reflecting & Evaluating | Collect and discuss quantitative and qualitative information about the success of implementation and/or the innovation  *Use this construct to capture themes related to Reflecting & Evaluating that are not included in the subconstructs below* |
| 1. Implementation | Collect and discuss quantitative and qualitative information about the success of implementation |
| 2. Innovation | Collect and discuss quantitative and qualitative information about the success of the innovation |
| I. Adapting | Modify the innovation and/or the Inner Setting for optimal fit and integration into work processes |
